# APBC 2010. The Eighth Asia Pacific Bioinformatics Conference Bangalore, India, 18-21 January 2010

**DOI:** 10.1186/1471-2105-11-S1-I1

**Published:** 2010-01-18

**Authors:** Laxmi Parida, Gene Myers

**Affiliations:** 1 IBM T J Watson Research, Yorktown, USA; 2 Howard Hughes Medical Institute, Janelia Farm, USA

## Introduction

The first Asia Pacific Bioinformatics Conference was held in 2003 in Adelaide, Australia, and has been an annual event ever since. While the locations of the previous conferences have been in the Pacific Rim, this is the first time that APBC is being held in South Asia. India is not just an emerging economy but also a burgeoning center for biotechnology and related sciences.

This ISCB affiliated event was well publicized through the web, various related mailing lists and carefully targeted poster displays. We received a healthy number of submissions from 31 countries: Netherlands, Denmark, Chile, Sweden, Poland, Saudi Arabia, Portugal, Philippines, Malaysia, France, Ireland, Iran, New Zealand, United Arab Emirates, Norway, Israel, United Kingdom, Italy, Switzerland, Australia, Singapore, Japan, Republic of Korea, Germany, Bangladesh, Canada, Hong Kong, Taiwan, China, United States and India. We are happy to observe that the participation covers all continents, with the exception of Africa. While the list here has been compiled using the location of the affiliated institutes, we are certain that the authors themselves represent a wider geography than this.

We received 231 submissions, about forty percent more than we expected (and surpassing each of the numbers in the previous editions of APBC). We have deliberately used a very flexible definition of "bioinformatics", as it is a continually evolving field, to define the scope of the conference. The diverse composition of the program committee (see Additional file 1) was well equipped to handle the challenge of the broad spectrum of topics covered under the large, versatile umbrella of bioinformatics and computational biology. This is reflected in the following categorization of the subject matter: (1) Patterns and Motifs, (2) Gene Regulation, (3) Interactomics, (4) Functional Genomics, (5) Genomics & Metagenomics, (6) Proteomics, (7) Disease Studies, (8) Association Studies, (9) Comparative Genomics, (10) Structural Genomics and (11) Phylogeny.

The program committee, working in all time-zones, did an excellent job, burning the midnight oil when required. We targeted about three independent reviews for each of the submissions. We received four reviews for 1 paper, two reviews for 49 papers and three reviews for each of the remaining. None of the papers with two reviews were close-calls. At the end of the day, we made the difficult decision of selecting 67 papers for oral presentation at the conference and publication in BMC Bioinformatics. We are cognizant of the fact that due to the typical constraints of a conference setting many worthy submissions did not make it to this short list. Judging by the diversity of the topics as well as the reviews, we have decided deliberately not to indulge in 'best paper' assignments. We are very grateful to the program committee members who invested a significant amount of time and energy, in spite of their busy schedules, to carry out their tasks in an objective and timely manner.

## Competing interests

The authors declare that they have no competing interests.

## Supplementary Material

Additional file 1 Program Committee. Click here for file

