# Supplementary material for: APBC 2010. The Eighth Asia Pacific Bioinformatics Conference Bangalore, India, 18-21 January 2010
Source: BMC Bioinformatics. 2010 Jan 18;11(Suppl 1):I1. doi: 10.1186/1471-2105-11-S1-I1 (PMC3397316; doi:10.1186/1471-2105-11-S1-I1)
Supplement: Additional file 1 — Program Committee. [file 1471-2105-11-S1-I1-S1.pdf]

APBC 2010

The Eighth Asia Pacific Bioinformatics Conference

Bangalore, India, 18-21 January 2010

Program Committee

Srinivas Aluru, Iowa State University, USA
Tatsuya Akutsu, Kyoto University, Japan
Joel Bader, Johns Hopkins University, USA
Chitta Baral, Arizona State University, USA
Upinder Bhalla, National Center for Biological Sciences, India
Alok Bhattacharya, Jawaharlal Nehru University, India
Harmen Bussemaker, Columbia University, USA
Nagasuma Chandra, IISc, Bangalore, India
Phoebe Chen, Deakin University, Australia
Francis Chin, Hong Kong University, Hong Kong
Nadia El-Mabrouk, University of Montreal, Canada
Mikhail Gelfand, Institute for Information Transmission Problems RAS, Russia
Roderic Guigo, Centre de Regulacio Genomica, Barcelona, Spain
Wen-Lian Hsu, Academia Sinica, Taiwan
Daniel Huson, University of Tubingen, Germany
Jenn-Kang Hwang, National Chiao Tung University, Taiwan
Uri Keich, University of Sydney, Australia
Tak Wah Lam, University of Hong Kong, Hong Kong
Doheon Lee, KAIST, Korea
Sang Yup Lee, KAIST, Korea
Ming Li, University of Waterloo, Canada
Wentian Li, Feinstein Institute for Medical Research, USA
Jingchu Luo, Peking University, China
Bin Ma, University of Waterloo, Canada
Hiroshi Mamitsuka, Kyoto University, Japan
Sharmila Mande, TCS, India
Shekhar Mande, CDFD, India
Bud Mishra, New York University, USA
Satoru Miyano, University of Tokyo, Japan
Debasisa Mohanty, National Institute of Immunology, India
Gene Myers, HHMI, Janelia Farm, USA (co-chair)
Kenta Nakai, University of Tokyo, Japan
Laxmi Parida, IBM T.J. Watson Research & NYU, USA (co-chair)
Gajendra Raghava, Institute of Microbial Technology, India
Mark Ragan, The University of Queensland, Australia

Naren Ramakrishnan, Virginia Tech, USA
Marie-France Sagot, INRIA and University Claude Bernard Lyon, France
R. Sankararamakrishnan, IIT Kanpur, India
David Sankoff, University of Ottawa, Canada
Rahul Siddharthan, The Institute of Mathematical Sciences, India
Steven Skiena, Stony Brook University, USA
Paul Spellman, Lawrence Berkeley National Laboratory, USA
Fengzhu Sun, University of Southern California, USA
Lusheng Wang, City University of Hong Kong, Hong Kong
Pramod Wangikar, IIT Bombay, India
Limsoon Wong, National University of Singapore, Singapore
Eric Xing, Carnegie Mellon University, USA
Louxin Zhang, National University of Singapore, Singapore
Michael Zhang, Cold Spring Harbor Laboratory, USA
Xuegong Zhang, Tsinghua University, China
Hongyu Zhao, Yale University, USA
